# Simultaneous Bilateral Rupture of the Triceps Tendon in a Renal Transplant Patient

**DOI:** 10.1155/2015/903690

**Published:** 2015-08-20

**Authors:** Ezequiel E. Zaidenberg, Gerardo L. Gallucci, Jorge G. Boretto, Pablo De Carli

**Affiliations:** Institute of Orthopaedics “Carlos E. Ottolenghi” Italian Hospital of Buenos Aires, C1199ACK Buenos Aires, Argentina

## Abstract

The unilateral rupture of the triceps brachii tendon is a rare lesion representing 1% of all tendon injuries. The most common causes are the result of a contraction against resistance (especially weightlifters) and direct trauma. It has also been associated with systemic diseases such as diabetes mellitus, chronic renal failure, secondary hyperparathyroidism, and use of systemic corticosteroids. Simultaneous bilateral rupture of the triceps tendons is less frequent and has been described in association with chronic metabolic disorders, especially in those patients on hemodialysis. This paper presents a case of bilateral triceps tendon rupture of a 36-year-old woman with renal transplantation secondary to chronic renal failure. Early surgical repair was performed using a bone tunnel technique with a nonabsorbable suture. Clinically active extension with 135 degrees of range of motion was achieved.

## 1. Introduction

The unilateral rupture of the triceps brachii tendon is a rare lesion representing 1% of all tendon injuries. The most common causes are the result of a contraction against resistance (especially weightlifters) and direct trauma [1]. It has also been associated with systemic diseases such as diabetes mellitus, chronic renal failure, secondary hyperparathyroidism, and use of systemic corticosteroids [2].

Acute, simultaneous bilateral complete rupture of the triceps tendons in patients with chronic renal failure is a rare injury; to our knowledge, only 3 cases have been reported in the international literature [3–5].

This paper describes a case of simultaneous bilateral triceps tendon rupture in a patient with renal transplantation secondary to chronic renal failure.

## 2. Case Report

A 36-year-old woman came to our emergency room with pain, massive effusions, and ecchymoses of both elbow joints. While going upstairs several hours earlier, she fell on her outstretched hands and experienced sudden, moderate pain and a popping sound in her left elbow followed by similar pain in her right elbow a few seconds later.

Physical examination revealed moderate pain and swelling in both elbows and a palpable dimple with tenderness just proximal to the olecranon. She was unable to extend her elbows against gravity even after adequate analgesic administration.

The patient suffered from chronic renal failure secondary to acute glomerulonephritis and had undergone maintenance hemodialysis 3 times a day for 12 years. She had received a kidney transplant three years before this trauma. She had been receiving 4 mg of prednisone and 720 mg of mycophenolate daily at the time of injury. She had also suffered patellar tendon rupture of both knees 5 years earlier and was surgically treated.

**Figure 1:**
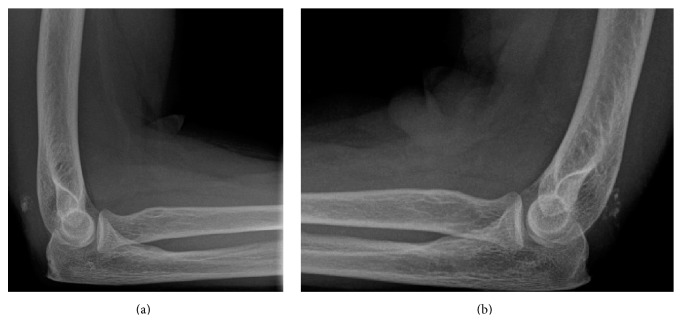
Lateral radiographs showing triceps tendon avulsion with a small piece of bone from the olecranon of the left (a) and right (b) elbow.

**Figure 2:**
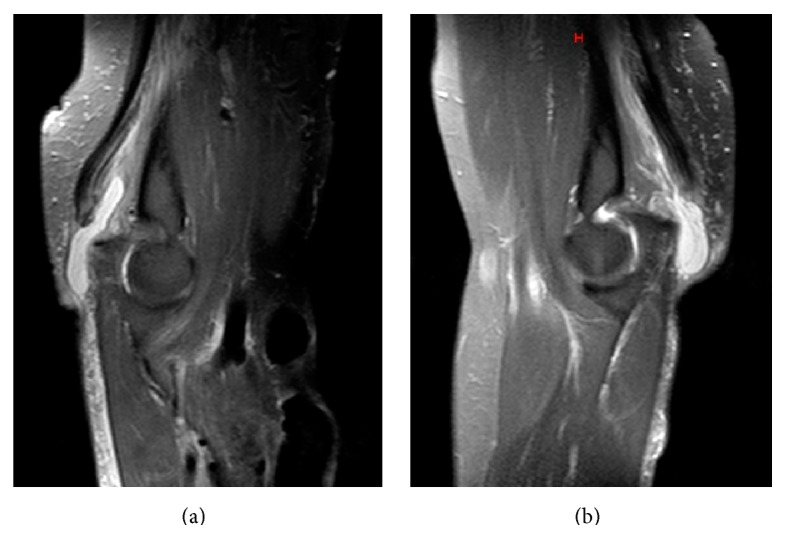
Magnetic resonance images of right (a) and left (b) elbows demonstrating complete avulsion of triceps tendon from the olecranon.

Radiographs revealed a small osseous flake just proximal to the olecranon in both arms and the absence of associated fractures (Figure 1). Magnetic resonance imaging confirmed the bilateral complete rupture of the triceps tendon (Figure 2).

The laboratory findings at the time of the injury include an estimated glomerular filtration rate of 40 mL/min, blood urea of 95 mg/mL (normal: 17–55 mg/mL), creatinine of 1.65 mg/mL (normal: 0.40–1.10 mg/mL), sodium of 139 mmol/L, potassium of 6.0 mmol/L, serum calcium of 10.9 mEq/L (normal: 4.5–5.4 mEq/I), serum phosphorus of 7.2 mEq/L (normal: 2–5.4 mEq/L), and parathormone (PTH) of 386 pg/mL (normal: 10–75 pg/mL). The above biochemical findings showed a moderate decrease in the glomerular filtration rate and a secondary hyperparathyroidism associated.

At operation, a complete and symmetrical triceps tendon avulsion with a small piece of bone from the olecranon was found bilaterally. Both triceps tendons appeared normal, other than being ruptured at their osteotendinous junctions.

**Figure 3:**
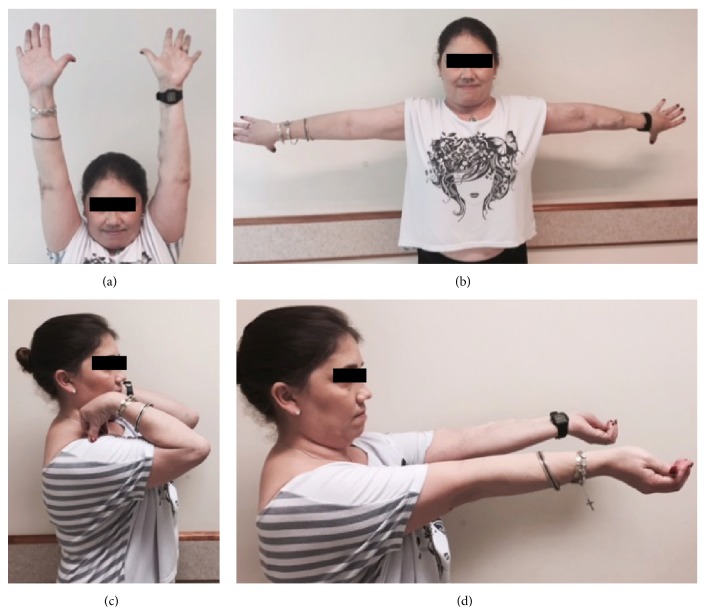
Clinical photograph showing elbow extension (a, b, and d) and flexion (c) at 3-month follow-up.

A primary repair was performed using nonabsorbable sutures passed through holes drilled in the olecranon with the technique described by Yeh et al. [6]. Postoperatively, the elbows were immobilized in a long-arm cast for 3 weeks, followed by progressive active flexion in a controlled motion brace. Active strengthening of the triceps was begun at about 6 weeks. Clinically active extension with 135 degrees of range of motion was achieved within 3 months (Figure 3).

At 12-month follow-up, the DASH score was 6 and the strength of extension of both elbows was M5 according to the British Medical Research Council (BMRC).

## 3. Discussion

Spontaneous bilateral rupture of the triceps tendons is uncommon and has been described in association with chronic metabolic disorders such as renal failure, especially in those patients on hemodialysis [7, 8]. Finlayson et al. described the association between the chronic acidosis in renal failure and the connective tissue elastosis [9]. Murphy and McPhee reported the rupture of several tendons in patients with chronic renal disease [10].

Spontaneous rupture of the tendon has also been reported in association with secondary hyperparathyroidism. In 1962, Preston and Adicoff suggested that deposits of calcium were the cause of weakening of the tendon tissue [11]. This mechanism can be explained by the effect of parathyroid hormone on bone matrix, increasing bone reabsorption, thus weakening the bone-tendon interface [12].

Other causes of spontaneous rupture were described in patients with corticosteroid treatment, abnormalities in collagen catabolism, lax tendons, metabolic acidosis, fluoroquinolone administration, and dialysis-associated amyloidosis [13].

Irby and Hume proposed that the tendon degeneration may also be a complication of renal transplantation resulting from a destructive posttransplant autoimmune response [14]. In addition, tendon ruptures are more frequent in individuals with kidney disease in dialysis or before kidney transplantation than in patients receiving other organ transplantations [15]. It is, therefore, more likely that tendon ruptures are related to metabolic changes associated with kidney disease rather than with transplantation or with glucocorticoid treatment per se.

We believe that, in an end-stage renal disease patient with a bilateral painful elbow and limited range of motion, the tendon rupture diagnostic must be considered, which can be missed, first diagnosed, and treated as being pseudogout or an inflammatory disease.

Early surgical management with repair of the tendon to bone can provide a satisfactory outcome.
